# VALD-3, a Schiff base ligand synthesized from o-vanillin derivatives, induces cell cycle arrest and apoptosis in breast cancer cells by inhibiting the Wnt/β-catenin pathway

**DOI:** 10.1038/s41598-021-94388-x

**Published:** 2021-07-22

**Authors:** Hongling Li, Chunyan Dang, Xiaohui Tai, Li Xue, Yuna Meng, Shuping Ma, Jing Zhang

**Affiliations:** Division of Oncology, Gansu Provincial Hospital, 204 Donggang West road, Chengguan district, Lanzhou City, 730000 Gansu Province China

**Keywords:** Cancer, Drug discovery, Oncology

## Abstract

Schiff base compounds and their metal complexes have become important synthetic organic drugs due to their extensive biological activities, which include anticancer, antibacterial and antiviral effects. In this study, we investigated the cytotoxic and apoptotic effects of VALD-3, a Schiff base ligand synthesized from o-vanillin derivatives, on human breast cancer cells and the possible underlying mechanisms. 3-(4,5-Dimethylthiazol-2-yl)-2,5-diphenyltetrazolium bromide (MTT)-test was used to observe the proliferation of human breast cancer MCF-7 and MDA-MB-231 cells induced by VALD-3. Flow cytometry analysis showed that VALD-3 triggered cell cycle arrest and induced apoptosis of breast cancer cells. Western blot analysis revealed that VALD-3 upregulated pro-apoptotic proteins (Bad and Bax), downregulated anti-apoptotic proteins (Bcl-2, Bcl-xl, survivin and XIAP) and increased the expression of cleaved caspase-3, cleaved caspase-8, Cyto-c and cleaved PARP. VALD-3 also regulated the Wnt/β-catenin signaling pathway in breast cancer cells, inhibiting the activation of downstream molecules. By xenografting human breast cancer cells into nude mice, we found that VALD-3 significantly suppressed tumor cell growth while showing low toxicity against major organs. In addition, survival analysis showed that VALD-3 can significantly prolong the survival time of mice (*P* = 0.036). This study is the first to show that VALD-3 induces apoptosis and cell cycle arrest in human breast cancer cells by suppressing Wnt/β-catenin signaling, indicating that it could be a potential drug for the treatment of breast cancer.

## Introduction

As in most other countries, breast cancer is the most common form of cancer in women in China, greatly impacting the national economy, social development and women's health. In 2019, the American Cancer Society (ACS) reported that breast cancer occupied the first place among the 10 leading cancer types in American women^1^. Age-standardized incidence rates for breast cancer in Chinese women showed a significant upward trend during the period 2000–2011^2^. Of note, breast cancer incidence in China continues to rise at a rate of 3–4% per year, which is twice the world average for the same period^3,4^. About 69,500 women died of breast cancer in China in 2015, and the mortality in urban and rural areas was 43.8 and 25.7 per thousand, respectively^2^.

Currently, the treatments for breast cancer mainly include surgery, chemotherapy, hormone therapy, targeted therapy and immunotherapy. Adjuvant chemotherapy has become the main treatment for breast cancer in China, and about 81.4% of patients with invasive breast cancer begin chemotherapy treatment^5^. Although the survival rate for breast cancer in China has greatly improved, the 5-year survival rate is still significantly lower than in Western developed countries. In addition, adverse drug reactions (emesis and diarrhea), disease recurrence, metastasis and drug resistance are persistent clinical problems^6^. Furthermore, breast cancer is one of the leading causes of catastrophic health expenditures in Chinese households due to low reimbursement and rising out-of-pocket expenses^6,7^. Therefore, it is imperative to develop new low-cost drugs with low toxicity to treat or prevent breast cancer.

Schiff bases are organic compounds that form coordination complexes with most metals. The study of Schiff base compounds and their metal complexes have become an important branch of synthetic organic chemistry due to their extensive biological activities, which include anticancer, antibacterial and antiviral effects^8,9^. Numerous studies have reported that Schiff base compounds can effectively eliminate stem cell-enriched cancer cells (HMLER-shEcad) and bulk cancer cells (HMLER)^10^, induce apoptosis to inhibit gastric tumor growth^11^, and significantly inhibit the growth of various human carcinoma cell lines derived from lung (A-549), breast (MDA-MB-231) and colon (LS174T)^12–14^.

There are many different types of Schiff base ligands: rigid, cyclic, or flexible. In recent years, there has been increased interest on soft ligands, mainly because flexible chain ligands are easy to synthesize, their structure can easily be adjusted, and the flexible chain can easily be modified and replaced, providing various coordination modes and enriching the types of complexes^15,16^. In our previous study, we found that Valdien, an o-vanillin-derived Schiff base ligand, showed anti-tumor effects in vivo and in vitro^17,18^. However, Valdien must be dissolved in DMSO and its water insolubility is one of the main disadvantages that limit its medicinal value. In addition, there are no studies on the effects of Valdien against human breast cancer, and its mechanism of action remains unknown. In many studies, for triple-negative breast cancer, the commonly used cell line is MDA-MB-231, while the non-triple negative cell line is MCF-7. In addition, studies have shown that Schiff bases with substituents on aldehydes show better antitumor effects than Schiff bases with substituents on amines^19^. Therefore, we synthesized three Schiff base ligands from o-vanillin derivatives designated as VALD-1, VALD-2 and VALD-3 by attaching the hydrophilic hydroxyl (–OH) group to o-vanillin to improve the water solubility of Schiff base ligands after reacting with diamines and polyamines, studying the effects on breast cancer cells. We found that VALD-3 significantly inhibited the proliferation of tumor cells.

The anti-tumor effects of VALD-3 against breast cancer cells and its specific molecular mechanisms were studied in vitro and in vivo. Moreover, we present evidence for the first time that Schiff base ligands from o-vanillin derivatives induce cell cycle arrest and apoptosis of human breast cancer cells.

## Materials and methods

### Chemicals and reagents

VALD-3 (purity ≥ 98.0%) was kindly supplied by Professor Song Pengfei (Northwest Normal University, China). Fetal bovine serum (FBS) was purchased from Lonser (Shanghai, China). Primary antibodies against Bax and Bad were obtained from Abcam (MA, USA), and against Bcl-2, cytochrome c, BCL-XL, caspase3, caspase8, PARP, XIAP, CyclinD1, CyclinB1, CDK-1 and β-actin were obtained from Proteintech Group Inc. (Chicago, IL, USA). Primary antibodies to probe the Wnt/β-catenin pathway were obtained from Cell Signaling Technology (Danvers, MA, USA). MTT (3-(4,5-dimethylthiazol-2-yl)-2,5-diphenyltetrazolium bromide), Hoechst 33258 fluorescent dye kit and RIPA cell buffer were obtained from Solarbio Co., Ltd (Beijing, China). The cell cycle and apoptosis kit were purchased from US EVERBRIGHT INC (UE, Suzhou, China). All other reagents and plastic material were obtained from commercial sources.

### Cell culture

Human breast cancer MCF-7 and MDA-MB-231 cell lines were purchased from the Stem Cell Bank, Chinese Academy of Sciences (Shanghai, China). Cells were cultured in RPMI-1640 medium (HyClone) containing 10% FBS and 1% penicillin streptomycin (HyClone) at 37℃ in a humidified 5% CO_2_ atmosphere.

### Xenograft of human breast cancer cells into nude mice

Six-week-old female BALB/c athymic nude mice (specific pathogen free, SPF) (18 ± 4 g) were purchased from Beijing Weitonglihua Laboratory Animal Co., Ltd. (Beijing, China; animal quality license, SCXK (Jing) 2016-0011). Mice were maintained in sterile conditions at a constant temperature of 22–24 °C, 50–55% humidity, and under a 12 h light/dark cycle. All methods were approved by the Institutional Animal Care and Use Committee of Gansu Provincial Hospital (No. 2017-015). The study was carried out in compliance with the ARRIVE guidelines and all experiments were approved and performed in accordance with the guidelines of Institutional Animal Care and Use Committee of Gansu Provincial Hospital.

MCF-7 tumor cells were resuspended at a density of 1.2 × 10^7^ cells/200 µL in saline solution were injected subcutaneously in the front armpit of the nude mice, followed by tumor growth measurements every 3 days. Negative controls were injected with 200 µL of phosphate buffered saline containing no cells. When the tumor size reached 100 mm^3^, 44 tumor-bearing mice were randomly assigned to the time course and survival experiments, which were conducted simultaneously. The time course experiment included five groups (negative control, control, 5 mg/kg/3d cisplatin, 20 mg/kg/d VALD-3 and 5 mg/kg/3d cisplatin + 20 mg/kg/d VALD-3, n = 8), whereas the survival experiment included two groups (control, 20 mg/kg/d VALD-3, n = 6). In the time course experiment, mice were sacrificed by cervical dislocation on day 13 and tumor tissues and blood samples were collected for further analysis. One mouse in the control group died on day 10 due to an oversized tumor. In the survival experiment, mice were treated until they died, the time of death of each mouse was recorded, and the survival analysis curve was plotted. During the course of the experiment, tumor size and body weight were measured every 3 days, and tumor volume was calculated based on the formula length × width^2^/2.

### MTT assay

The effects of VALD-3 on MCF-7 and MDA-MB-231 cell proliferation were measured with the MTT assay. MTT assay according to our previous test method^18^. Briefly, VALD-3 was dissolved in RPMI-1640 culture media at final concentrations of 0, 2.5, 5, 10, 20, and 40 mg/L. MCF-7 and MDA-MB-231 cells were seeded onto 96-well plates at a density of 3 × 10^4^ cells /well, incubated at 37℃for 24 h and then treated with various concentrations of VALD-3 (0, 2.5, 5, 10, 20, and 40 mg/L) for 24, 48 and 72 h. After treatment, 20 µl of 3-(4,5-dimethylthiazol-2-yl)-2,5-diphenyl-tetrazolium bromide (MTT, 5 mg/ml) was added to each well and cells were incubated for another four hours. Finally, 150 µl of DMSO was added to dissolve the formazan crystals. The absorbance at 570 nm was measured with a microplate reader (Molecular Devices, Sunnyvale, USA).

### Hoechst 33258 staining

Cells were incubated in 6-well plates, treated for 48 h with different concentrations of VALD-3 (0, 2.5, 5, 10, 20, 40 mg/L), washed with PBS and stained with Hoechst 33258 solution in the dark for about 5 min. Stained cells were observed and photographed with an inverted fluorescence microscope (Leica Microsystems, Wetzlar, Germany).

### Cell cycle analysis

The effects of VALD-3 on the cell cycle were analyzed in MCF-7 and MDA-MB-231 cells by PI single staining. Briefly, cells were seeded onto 6-well plates. After overnight incubation, cells were treated for 24 h with different concentrations of VALD-3 (0, 2.5, 5, 10, 20, 40 mg/L). Next, cells were collected and fixed at 4 °C overnight with pre-cooled 70% ethanol. The ethanol solution was discarded and the cells were washed twice with PBS. The proportion of DNA in each sample was determined using a cell cycle kit (Cat. No. C6031). Data were acquired on a BD FACS Calibur (BD Bioscience, USA) machine and analyzed using ModFit LT 3.2 software.

### Annexin V/PI staining assay

MCF-7 and MDA-MB-231 cells were seeded onto 6-well plates and treated with VALD-3 (0–40 mg/L) for 24, 48, and 72 h. The method was carried out according to the instructions of the Annexin V-FITC Apoptosis Assay Kit. Cells were then collected and washed twice with cold PBS. Next, cells were resuspended in 100 μL of 1 × binding buffer and incubated for 15 min at room temperature in the dark with 5 μL of annexin V and 5 μL of PI solutions (Annexin V-FITC Apoptosis Assay Kit, BD Biosciences, USA). The percent of apoptotic cells was determined by flow cytometry.

### Quantitative real-time RT-PCR (qRT-PCR)

Expression of Bcl-2, Bax, Wnt/β-catenin signaling pathway and its downstream target genes c-Myc and CyclinD1 was assessed by RT-PCR after VALD-3 (0–20 mg/L) treatment. The primers were designed and manufactured by Takara Bio with the following sequences: Bcl-2 (forward: 5′-GGATTGTGGCCTTCTTTGAG-3′, reverse: 5′-TACCCAGCCTCCGTTATCCT-3′), Bax (forward: 5′-CCGATTCATCTACCCTGCTG-3′, reverse: 5′-TGAGCCAATTCCAGAGGCAGT-3′), β-catenin (forward: 5′-CTT ACA CCC ACC ATC CCA CT-3′, reverse: 5′-CCTCCACAAATTGCTGCTGT-3′), c-Myc (forward: 5′-GCTGCTTAGACGCTGGATTT-3′, reverse: 5′-GGCATTCGACTCATCTCAGC-3′), cyclinD1 (forward: 5′-GCCGAATTCATGGAACACCAGCT-3′, reverse: 5′-TGCACCTGTAGACTGAGCTCGC-3′), and β-actin (forward: 5′-GGACTTCGAGCAAGAGATGG-3′, reverse: 5′-AGCACTGTGTTGGCGTACAG-3′). The RT-PCR reaction was performed using the SYBR green detection system (Takara, Japan) and the data were analyzed by the 2^−ΔΔCt^ method.

### Western blot analysis

MCF-7 and MDA-MB-231 cells were cultured and treated with VALD-3 (0, 5, 10, 20, 40 mg/L). After 48 h, cells were harvested and lysed with lysis buffer (PARP) containing 1% PMSF at 4 ℃ for 60 min. The protein concentration was quantified with a BCA Protein Assay kit (Solarbio, Beijing, China). Samples were mixed with loading buffer and boiled at 100 °C for 10 min. Proteins were separated by SDS-PAGE and transferred to polyvinylidene fluoride (PVDF) membranes. The PVDF membranes were blocked with TBST containing 5% skim milk for 1.5 h at room temperature under constant agitation. Using β-actin as an internal reference, followed by overnight incubation with the primary antibodies against Bax (Cat. No. ab32503), Bad (Cat. No. ab32445) and β-actin (Cat. No. 60008-1-AP) diluted at 1:5000, β-catenin (Cat. No. GB11015), Wnt/β-catenin pathway (Cat. No. 8655), caspase3 (Cat. No. 19677-1-AP), caspase8 (Cat. No. 13423-1-AP), cytochrome *c* (Cat. No. 10993-1-AP), and PARP (Cat. No. 13371-1-AP) diluted at 1:1000, Bcl-2 (Cat. No. 26967-1-AP), XIAP (Cat. No. 10037-1-AP) and Survivin (Cat. No. 10508-1-AP) diluted at 1:2000, CDK1 (Cat. No. 10762-1-AP) and CyclinB1 (55004-1-AP) at 4 °C and three washes with TBST for 10 min. Membranes were then incubated with the secondary antibodies for another 1.0 h at room temperature. After additional washes, protein bands were visualized by enhanced chemiluminescence (Santa Cruz Biotechnology, Santa Cruz, CA, USA). Quantification of protein bands was achieved by densitometric analysis using Image-Lab software.

### Histopathology and immunohistochemistry

Organs and tumors were fixed with 4% paraformaldehyde and embedded in paraffin. The paraffin-embedded specimens were cut into 4 µm thick slides and stained with hematoxylin and eosin (H&E) for pathological analysis. Tumor tissues were immunostained with antibodies specific for Bax and Bad. Images were captured using a light microscope (Nikon, Japan).

### TUNEL assay

Apoptotic cells in breast tumor tissue sections were detected using TUNEL staining, following the manufacturer’s instructions. The apoptotic cells were observed using a fluorescent microscope.

### Statistical analysis

Data were analyzed by one-way analysis of variance (ANOVA) and all results are expressed as the mean ± standard deviation. Differences among the treatment groups were assessed by LSD-*t* test, using Graph Pad Prism version 5.0 (Graph Pad Software Inc, CA, and USA). A value of *p* less than 0.05 was considered statistically significant.

## Results

### Cytotoxic and anti-proliferative in vitro effects of VALD-3 on breast cancer cells

**Figure 1:**
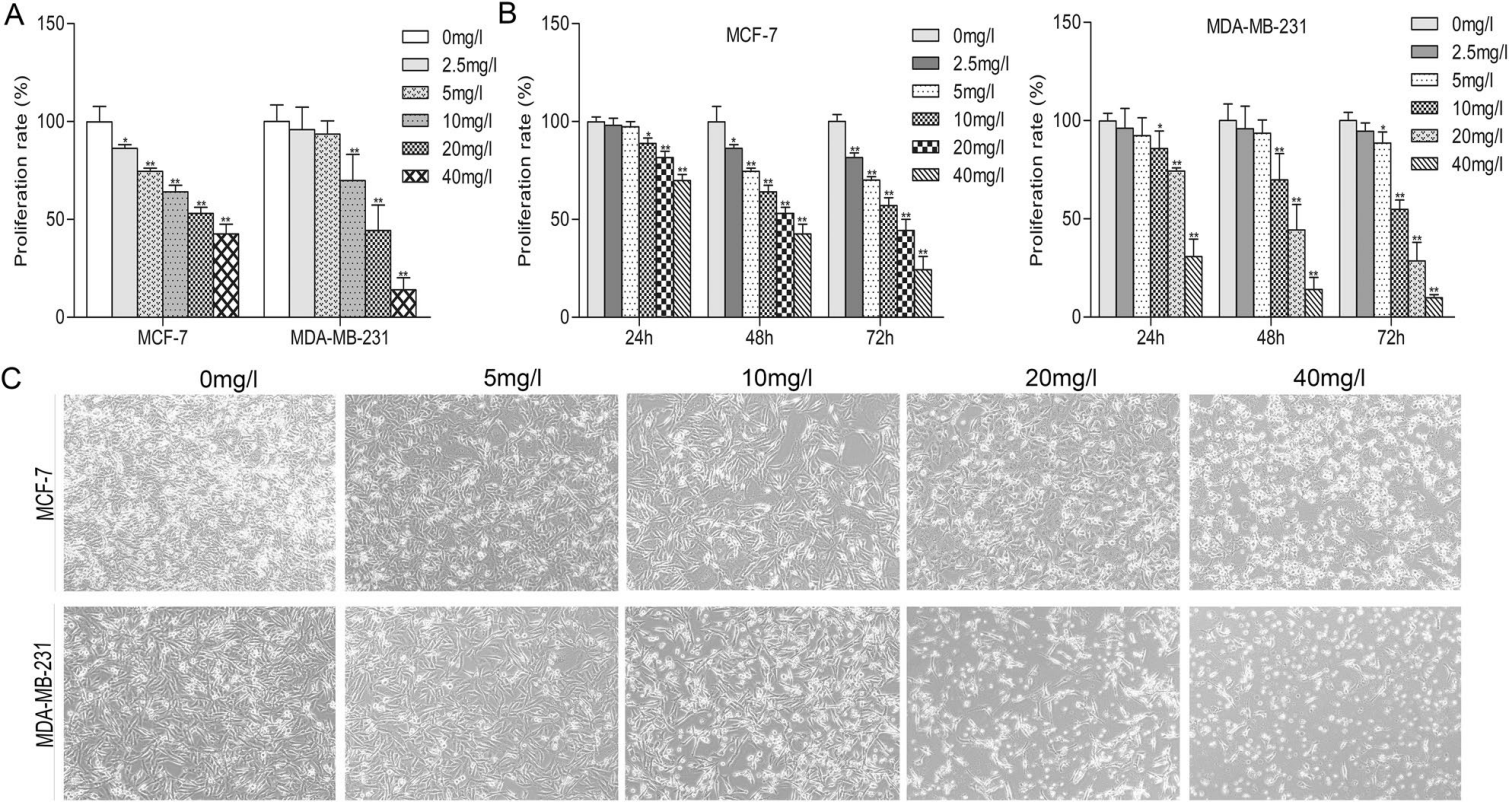
Effects of VALD-3 on the cytotoxicity and proliferation of breast cancer cell lines. (**A**) The effect of anti-proliferation of VALD-3 on MCF-7 and MDA-MB-231 cells was measured by MTT assay. Cells were inoculated into 96-wellplate and treated with different concentrations of VALD-3 for 48 h. (**B**) VALD-3 inhibits proliferation of MCF-7 and MDA-MB-231 cells in a concentration-dependent manner and a time-dependent manner. MCF-7 and MDA-MB-231 cells were cultured in 96-well plates and treated with different concentrations of VALD-3 for 24 h, 48 h and 72 h, respectively; the cell viability was determined by the MTT assay. Each data are presented as the mean ± SD from four independent experiments *P < 0.05, **P < 0.01 versus control group. (**C**) Morphological assessment of control and VALD-3 treated breast cancer MCF-7 and MDA-MB-231 cells.

MCF-7 and MDA-MB-231 cells were treated with different concentrations (2.5, 5, 10, 20 and 40 mg/l) of VALD-3 for 24, 48 and 72 h. The MTT assay was used to assess the effects of VALD-3 on cell viability and proliferation. The results showed that VALD-3 markedly inhibited MCF-7 and MDA-MB-231 proliferation after 48 h (Fig. 1A). As shown in Fig. 1B, VALD-3 significantly reduced the proliferation of MCF-7 and MDA-MB-231 cells in a dose- and time-dependent manner. Specifically, treatment with 40 mg/l of VALD-3 for 72 h significantly reduced MCF-7 cell viability to only 24.01 ± 5.87% of control. In the case of MDA-MB-231, cell viability was significantly reduced to only 9.69 ± 1.31% of control. To visually analyze the inhibitory effect, the cell morphology was observed under an inverted microscope after treating with VALD-3 for 48 h. In the VALD-3-treated groups, the number of breast cancer cells was significantly less than in the control group. Particularly, when the concentration of VALD-3 reached 10 mg/l, the cells contracted, became deformed and detached (Fig. 1C).

### VALD-3 induces apoptosis of MCF-7 and MDA-MB-231 cells

**Figure 2:**
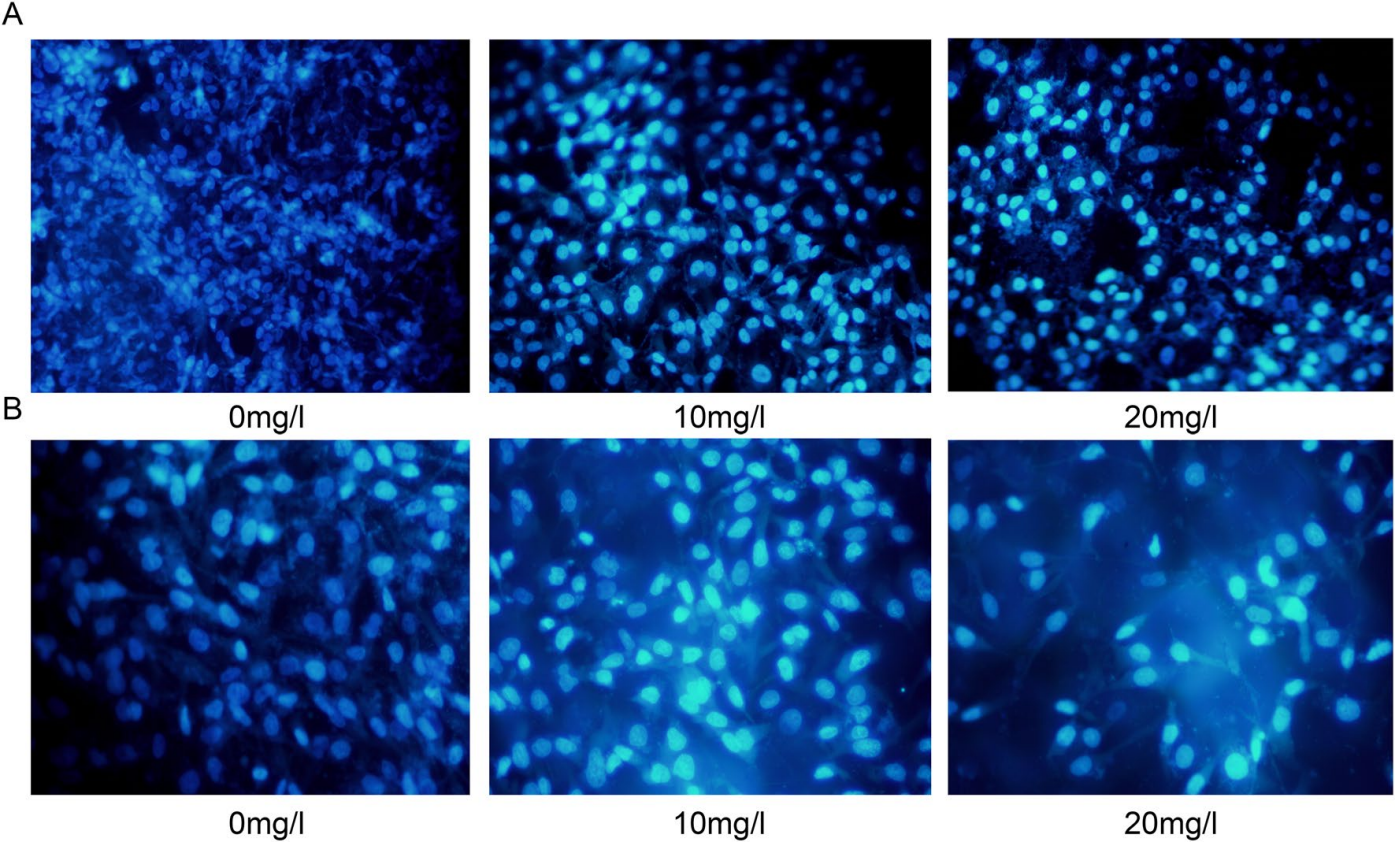
VALD-3 induces cell apoptosis in MCF-7 cells and MDA-MB-231 cells. Cells were treated without or with 10 mg/L, 20 mg/L VALD-3 for 48 h. Morphologic changes of MCF-7 cell (**A**) and MDA-MB-231 cells (**B**) VALD-3-treated were observed after Hoechst 33258 staining.

**Figure 3:**
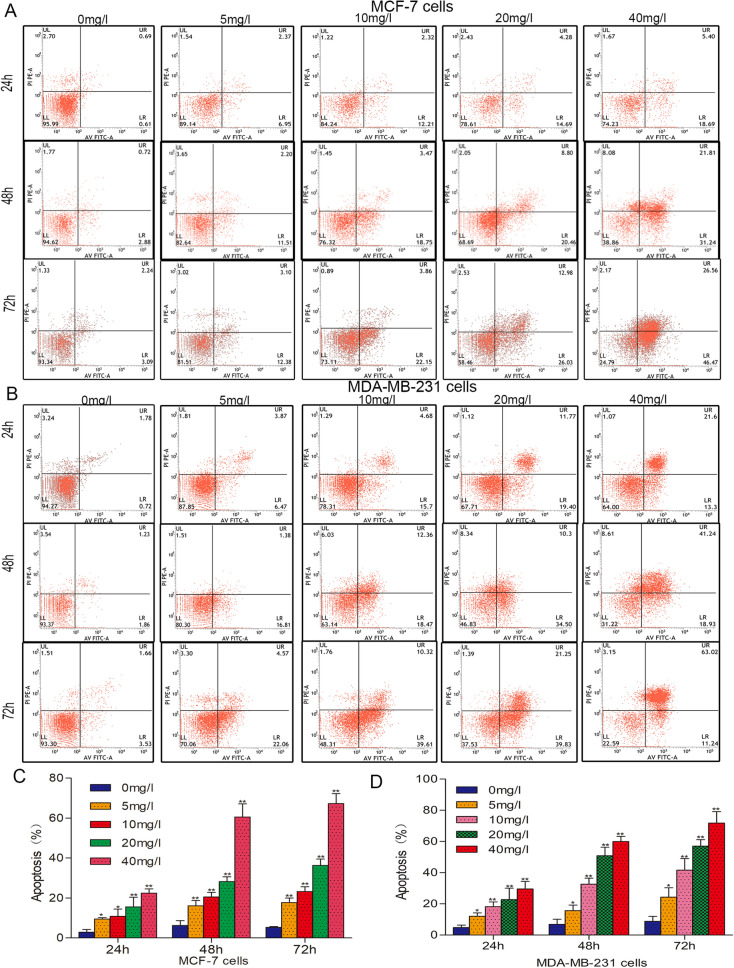
VALD-3 induces apoptosis in MCF-7 cells and MDA-MB-231cells. Apoptosis was analyzed via flow cytometry after Annexin V/PI staining. (**A**) Annexin V-FITC/PI double staining was used to detect the apoptotic rate of MCF-7 cells after VALD-3 treatment for 24 h, 48 h and 72 h. (**B**) Annexin V-FITC/PI double staining was used to detect the apoptotic rate of MDA-MB-231 cells after VALD-3 treatment for 24 h, 48 h, and 72 h. (**C**) Statistical analysis of the apoptotic rate of MCF-7 cell population after VALD-3 treatment for 24 h, 48 h and 72 h. (**D**) Statistical analysis of the apoptotic rate of MDA-MB-231 cell population after VALD-3 treatment for 24 h, 48 h and 72 h. *P < 0.05, **P < 0.01 compared with the control group. All data are presented as the mean ± SD from three independent experiments.

Hoechst 33258 staining was performed to determine if VALD-3 induced apoptosis of MCF-7 and MDA-MB-231 cells. The results showed that both cell lines exhibited evident apoptotic features after being treated with VALD-3, including nuclear fragmentation, irregular chromatin condensation and apoptotic body formation (Fig. 2A, B). Annexin V/PI staining was performed to quantify apoptosis. Flow cytometry analysis showed that the percentage of apoptotic MCF-7 cells increased significantly after treatment with VALD-3 for 24 h, reaching 24.09% at 40 mg/l (Fig. 3A). Treating for 48 h, 72 h with the same concentration of VALD-3 resulted in a significantly higher cell apoptosis rate than when the treatment was for 24 h (Fig. 3C). Similarly, the percentage of apoptotic MDA-MB-231 cells increased significantly with increasing VALD-3 concentrations when compared with control (Fig. 3B, D). These results indicate that VALD-3 induces apoptosis of breast cancer cells in a dose- and time-dependent manner.

### VALD-3 induces cell cycle arrest in MCF-7 and MDA-MB-231 cells.

**Figure 4:**
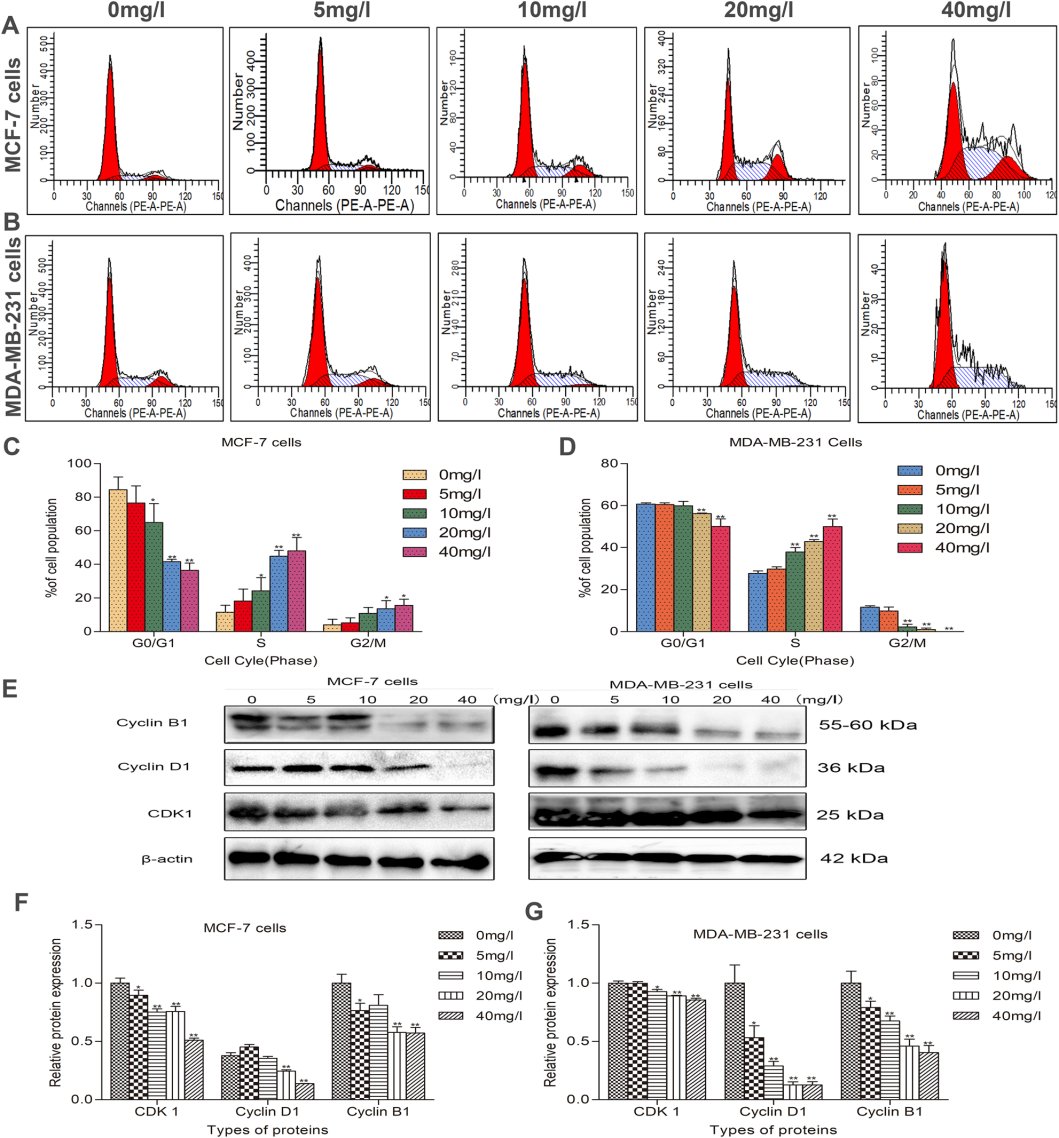
VALD-3 induced S and G2/M phase cell cycle arrest in MCF-7 cells and S phase cell cycle arrest in MDA-MB-231cells. Flow cytometry results showed that the cell cycle distribution of MCF-7cells (**A**) and MDA-MB-231cells (**B**) treated with VALD-3 for 48 h. (**C**) Percentages of cell population in each cell-cycle phase were analyzed in MCF-7cells (**C**) and MDA-MB-231cells (**D**) treated with VALD-3 for 48 h. (**E**) Levels of cell cycle related proteins CDK1, cyclinB1 and cyclinD1 proteins in VALD-3-treated breast cancer cells for 48 h were detected by western blotting. (**F**–**G**) The relative protein levels of CDK1, cyclinB1 and cyclinD1 proteins in VALD-3-treated breast cancer cells were analyzed. *P < 0.05, **P < 0.01 compared with the control group. All data are presented as the mean ± SD from three independent experiments.

To further examine the possible mechanisms underlying the anti-proliferative effects of VALD-3, the percentages of cells in each cell-cycle phase were measured by flow cytometry. As shown in Fig. 4A, VALD-3 induced cell cycle arrest in the S and G2/M phases in MCF-7 cells. The percentage of cells in the S phase increased from (11.51% ± 3.44%) to (45.98 ± 6.49)% and the percentage of cells in the G2/M phase increased from (4.02% ± 2.73%) to (15.63 ± 2.99)% (*P < 0.05) after treating with 40 mg/l VALD-3 for 48 h (Fig. 4C). Similarly, as shown in Fig. 4B, the percentage of VALD-3-treated MDA-MB-231 cells in the S phase was significantly higher than in the control group and the percentages of cells in the G0/G1 and G2/M phases were effectively reduced. VALD-3 induced cell cycle arrest in the S phase in MDA-MB-231 cells, increasing the percentage of S phase cells by (49.96 ± 2.95) % (*P < 0.05) (Fig. 4D). Western blots were also performed to examine the levels of cell cycle-related proteins (CDK1, Cyclin B and Cyclin D). Compared with the control group, the expression levels of CDK1, Cyclin B and Cyclin D in VALD-3-treated MCF-7 and MDA-MB-231 cells were significantly reduced (Fig. 4E, F, G). These results suggest that VALD-3 inhibits cell proliferation by regulating cell cycle-related proteins, inducing cell cycle arrest.

### VALD-3 regulates apoptosis-related proteins in MCF-7 and MDA-MB-231 cells

**Figure 5:**
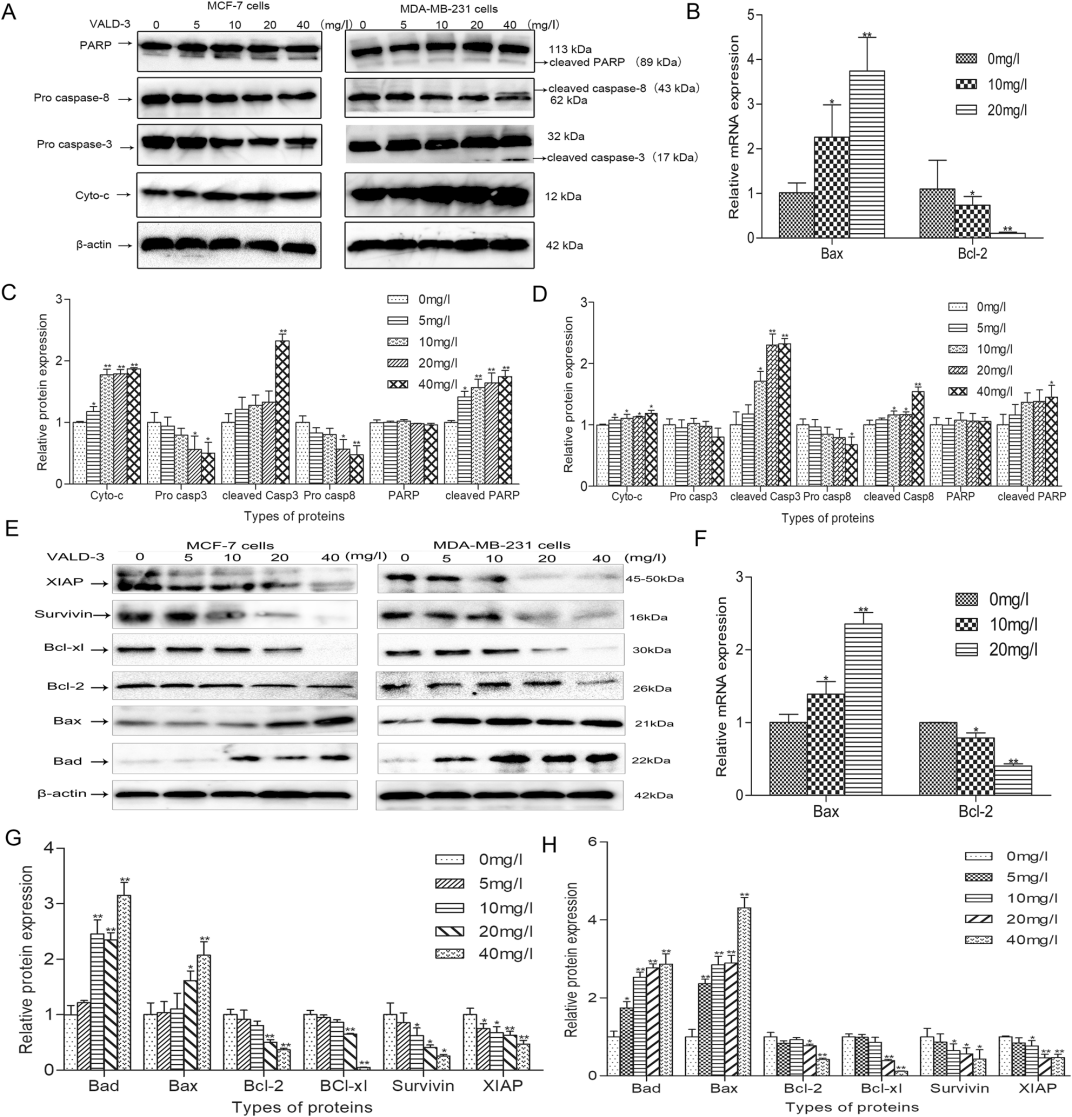
Effects of VALD-3 on the levels of apoptosis-related molecules in MCF-7 and MDA-MB-231 cells. (**A**) MCF-7cells and MDA-MB-231 cells were treated with the indicated concentrations of VALD-3 for 48 h, and levels of caspase-8, cleaved caspase-8, caspase-3, cleaved caspase-3, cytochrome *C*, PARP and cleaved PARP in VALD-3-treated breast cancer cells were detected by Western blotting. (**B**) The mRNA expression levels of MCF-7 cells were analyzed by qRT-PCR. (**C**) The relative levels of caspase-8, cleaved caspase-8, caspase-3, cleaved caspase-3, cytochrome *C*, PARP and cleaved PARP in MCF-7 cells were analyzed. (**D**) The relative levels of caspase-8, cleaved caspase-8, caspase-3, cleaved caspase-3, cytochrome *C*, PARP and cleaved PARP in MDA-MB-231 cells were analyzed. (**E**) MCF-7cells and MDA-MB-231cells were treated with the indicated concentrations of VALD-3 for 48 h, and expression of pro-apoptotic proteins and anti-apoptotic proteins in VALD-3-treated breast cancer cells were detected by Western blotting. (**F**) The mRNA expression levels of MDA-MB-231 cells were analyzed by qRT-PCR. (**G**) The relative levels of pro-apoptotic proteins and anti-apoptotic proteins in MCF-7 cells were analyzed. (**H**) The relative levels of pro-apoptotic proteins and anti-apoptotic proteins in MDA-MB-231 cells were analyzed. *P < 0.05, **P < 0.01 compared with the control group. All data are presented as the mean ± SD from three independent experiments.

The expression of proteins involved in apoptosis was investigated by Western blot using lysates of MCF-7 and MDA-MB-231 cells. Compared with the control group, the levels of cleaved Caspase-3, cleaved Caspase-8 and cleaved PARP were significantly increased in VALD-3-treated breast cancer cells, indicating that VALD-3 could simultaneously activate these proteins (Fig. 5A, C, D). Furthermore, the levels of Cytochrome *C*, a molecule upstream of Caspase-9, were examined by Western blot. The results showed that the levels of Cytochrome *C* were markedly higher than in the control group (Fig. 5A). These results indicate that VALD-3 triggers MCF-7 and MDA-MB-231 apoptosis by a mitochondrial-mediated apoptotic signaling pathway.

### VALD-3 promotes the expression of pro-apoptotic proteins and inhibits the expression of anti-apoptotic proteins in MCF-7 and MDA-MB-231 cells

To verify that apoptosis of MCF-7 and MDA-MB-231 cells induced by VALD-3 was related to activation of the mitochondrial apoptotic pathway, we measured the levels of Bad, Bax, Bcl-2, Bcl-xl, Survivin and XIAP by Western blot. We found that the levels of pro-apoptotic Bad and Bax proteins were significantly increased. In contrast, the levels of anti-apoptotic proteins like Bcl-2, Bcl-xl, Survivin and XIAP, were markedly reduced in VALD-3-treated MCF-7 and MDA-MB-231 cells when compared with control cells (Fig. 5E). As shown in Fig. 5G, H, analyses of the relative levels of pro-apoptotic and anti-apoptotic proteins were consistent with the above results. In addition, Bcl-2 and Bad mRNA expression levels in MCF-7 cells and MDA-MB-231 cells were analyzed by qRT-PCR. The results showed that VALD-3 downregulated Bcl-2 and upregulated Bax expression at the mRNA level (Fig. 5B, F).

Finally, immunohistochemistry tests were performed to detect the expression of Bad and Bax in tumor tissues. The results showed that treatment with VALD-3 significantly increased the expression of Bad and Bax in tumor tissues (Fig. 7I). These results demonstrate that VALD-3 triggers apoptosis via a caspase-dependent intrinsic pathway.

### VALD-3 modulates the Wnt/β-catenin pathway and downstream molecules in MCF-7 and MDA-MB-231 cells

**Figure 6:**
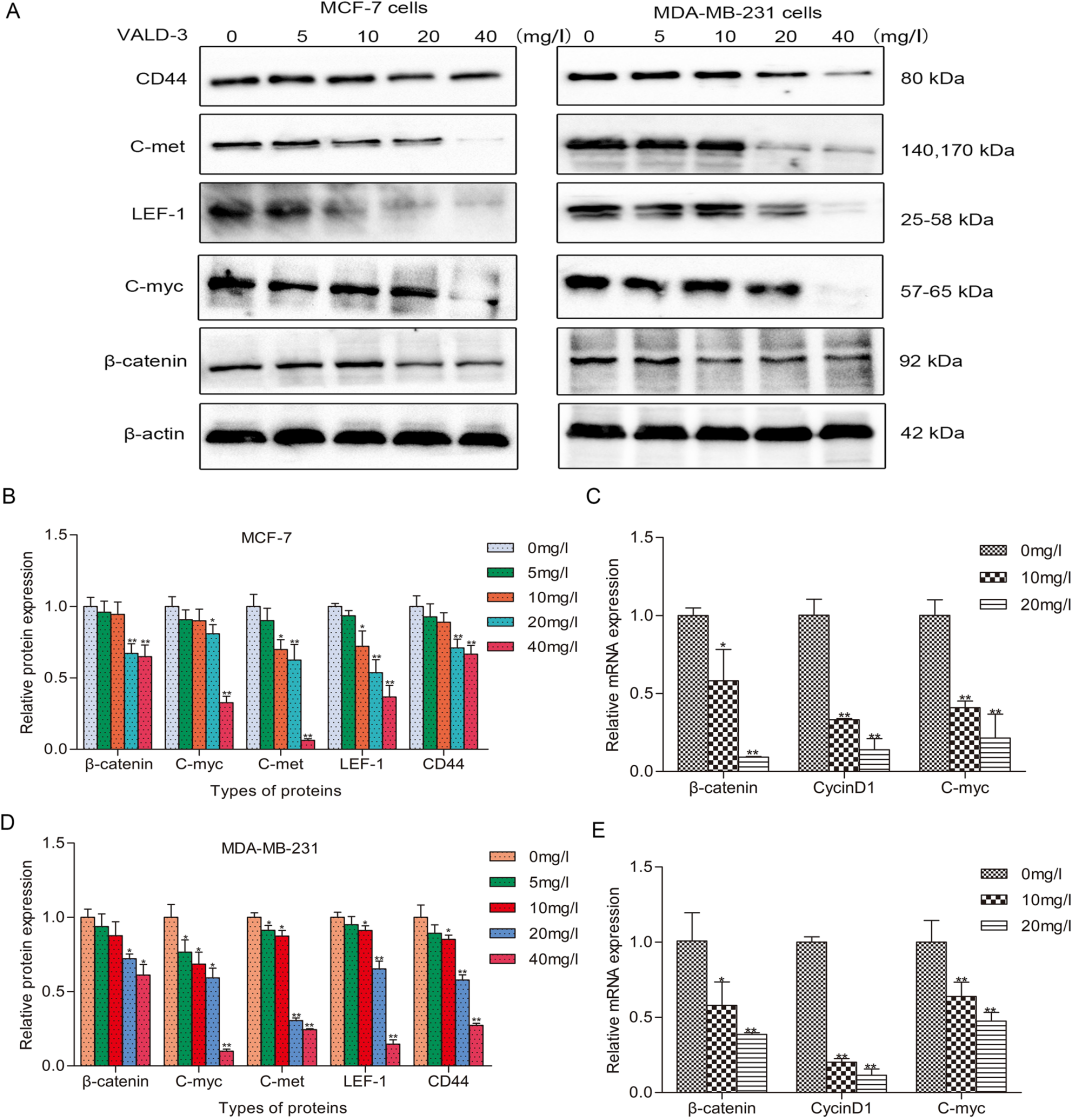
Effects of VALD-3 on the levels of Wnt/β-catenin pathway and its downstream molecules in MCF-7 cells and MDA-MB-231 cells. (**A**) MCF-7cells and MDA-MB-231cells were treated with the indicated concentrations of VALD-3 for 48 h, and levels of β-catenin, c-myc, LEF-1, c-met and CD44 in VALD-3-treated breast cancer cells were detected by Western blotting. (**B**) The relative levels of β-catenin, c-myc, LEF-1, c-met and CD44 in MCF-7 cells were analyzed. (**C**) The mRNA expression levels of β-catenin, c-myc and cyclinD1 in MCF-7 cells were analyzed by qRT-PCR. (**D**) The relative levels of β-catenin, c-myc, LEF-1, c-met and CD44 in MDA-MB-231 cells were analyzed. (**E**) The mRNA expression levels of β-catenin, c-myc and cyclinD1 in MDA-MB-231 cells were analyzed by qRT-PCR. *P < 0.05, **P < 0.01 compared with the control group. All data are presented as the mean ± SD from three independent experiments.

The Wnt/β-catenin pathway is closely linked to the development of breast cancer, so we examined the levels of some of its signaling components and downstream molecules in VALD-3-treated MCF-7 and MDA-MB-231 cells. Western blot analysis showed that the levels of β-catenin, C-myc, LEF-1, C-met and CD44 in VALD-3-treated MCF-7 and MDA-MB-231 cells were significantly lower (Fig. 6A). Analysis of the relative protein levels showed that VALD-3 inhibited the Wnt/β-catenin pathway and its downstream molecules in a concentration dependent manner (Fig. 6B, D). In addition, qRT-PCR analysis showed that VALD-3 reduced β-catenin, C-myc and CyclinD1 mRNA levels in MCF-7 and MDA-MB-231 cells (Fig. 6C, E).

### VALD-3 reduced tumor growth in an MCF-7 tumor xenograft model

**Figure 7:**
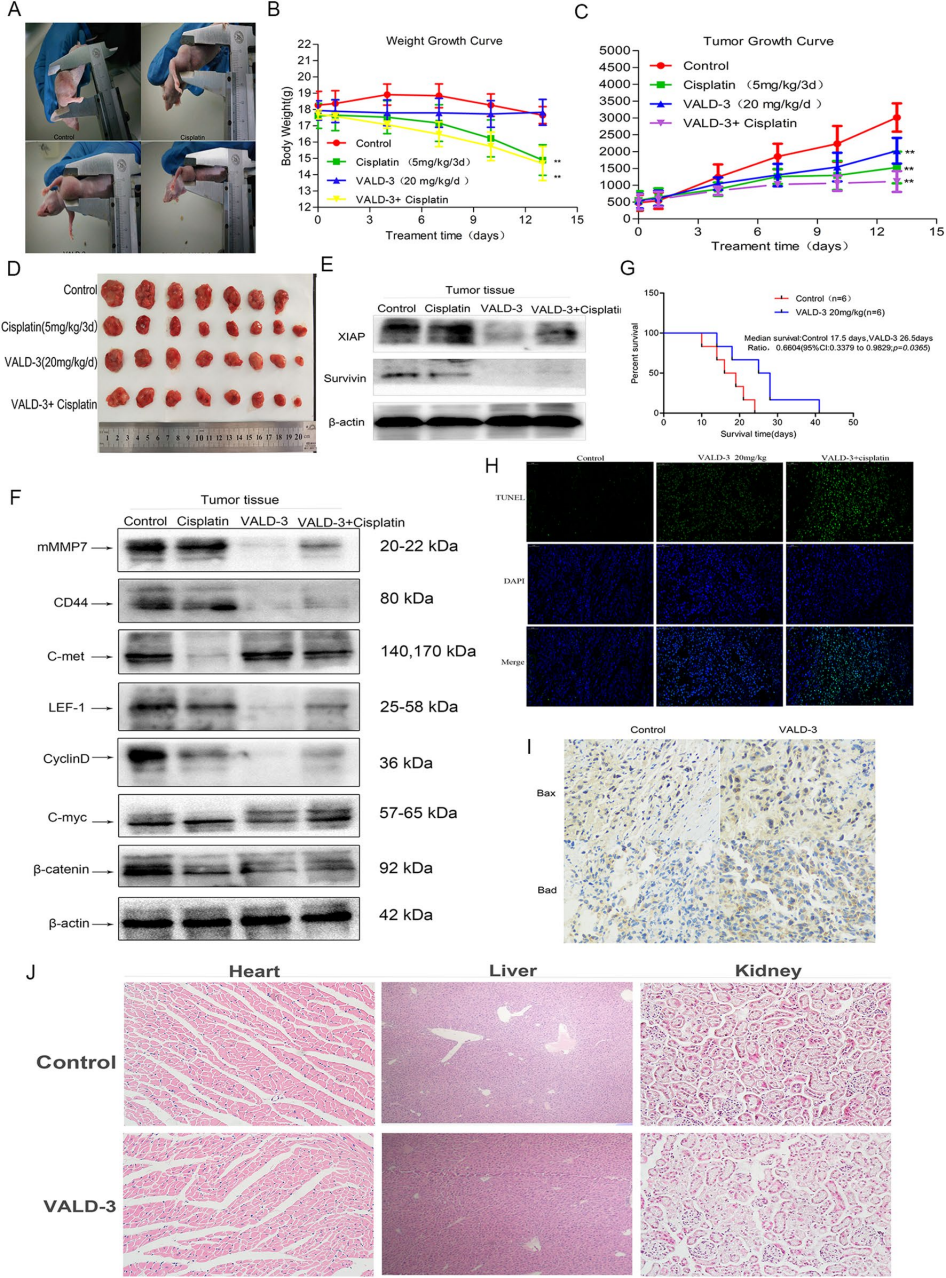
Effects of VALD-3 on the anti-tumor in vivo. (**A**) Tumor formation in nude mice. (**B**) Body weight were measured every 3 days. (**C**) Tumor volume variation, **p < 0.05 versus control group. Each point represents the mean ± SD. (**D**) Representative images of MCF-7 xenograft tumors isolated from the control group, cisplatin (5 mg/kg/3d), VALD-3-treated groups and VALD-3 + cisplatin-treated group after treatment for 13 days. (**E**) Western blotting were performed to analyze the expression levels of Wnt/β-catenin pathway and its downstream molecules and (**F**) the expression levels of Surviving and XIAP in tumor tissues. (**G**) Survival analysis curve. (**H**) The apoptotic levels of human breast tumor tissues were measured by TdT-mediated dUTP-biotin nick end labeling (TUNEL) staining. (**I**) Immunohistochemical analysis of Bad and BAX in tumor tissues. Pretreatment with VALD-3. (**J**) HE staining of tumor specimens and major organs. Scale bars = 100 µm.

An MCF-7 tumor xenograft model was established in nude mice to evaluate whether VALD-3 inhibited the growth of breast cancer cells in vivo. As illustrated in Fig. 7C, the cisplatin (5 mg/kg/3d), VALD-3 (20 mg/kg) and VALD-3 + cisplatin groups all showed significant inhibition of tumor growth when compared with the control group. It was observed from Fig. 7A that the size of the xenograft of the treatment group is significantly smaller than that of the control group. The percent inhibitions were 57.4% for cisplatin (5 mg/kg/3d), 51.0% for VALD-3 (20 mg/kg/d) and 58.2% for VALD-3 + cisplatin (Fig. 7D; Table1). In addition, the weight curve showed important differences between the control and experimental groups. As shown in Fig. 7B, the body weights of mice belonging to the cisplatin (5 mg/kg/3d) and VALD-3 + cisplatin groups were significantly lower than the control. Importantly, whereas the body weights of mice in the cisplatin group were lower than in the control group, we observed no significant differences in body weights between the VALD-3 (20 mg/kg/d) and control groups. On the day of sacrifice, the body weights for the cisplatin, VALD-3, VALD-3 + cisplatin and control groups were 14.90 ± 0.87 g, 17.83 ± 0.73 g, 14.69 ± 0.97 g and 17.66 ± 0.48 g, respectively (Table 1). Western blots were also performed to examine the levels of β-catenin and downstream molecules. Compared with the control group, the expression levels of β-catenin, c-myc, cyclinD, LEF-1, CD44 and Mmmp7 in VALD-3 (20 mg/kg/d) group were significantly reduced (Fig. 7F). The results of Western blots also showed the levels of apoptosis related protein (XIAP and Survivin) significantly down-regulated in VALD-3 (20 mg/kg/d) tumor tissues (Fig. 7E) and the increased cellular apoptosis were confirmed by TUNEL staining (Fig. 7H). In addition, survival analysis results showed that VALD-3 can significantly prolong the survival time of mice (*P* = 0.036) (Fig. 7G).

**Table 1:** Tumor growth parameters of VALD-3 treatment on the breast cancer xenografts model.

Group	Animal weight		Tumor volume		Tumor weight (g)	Inhibition rate (IR) (%)
	Initial (g)	Sacrificed (g)	Initial (cm^3^)	Sacrificed (cm^3^)		
Control	18.27 ± 0.77	17.66 ± 0.48	0.48 ± 0.22	3.01 ± 0.39	2.32 ± 0.53	0.00
Cisplatin (5 mg/kg/3d)	17.61 ± 0.71	14.90 ± 0.87	0.55 ± 0.25	1.52 ± 0.44	0.99 ± 0.44*	57.40
VALD-3 (20 mg/kg/d)	17.94 ± 0.55	17.83 ± 0.73	0.53 ± 0.20	2.03 ± 0.35	1.14 ± 0.30*	51.00
VALD-3 + Cisplatin	17.97 ± 0.34	14.69 ± 0.97	0.51 ± 0.21	1.11 ± 0.29	0.97 ± 0.36*	58.20

*P < 0.05 compared with the control group with significant differences.

Furthermore, H&E staining showed that VALD-3 at a concentration of 20 mg/kg did not induce significant heart, hepatic or kidney damage when compared with control (Fig. 7J). These results suggest that VALD-3 has potent anti-breast cancer effects but with low toxicity.

## Discussion

Schiff bases are mainly organic compounds containing imine or methylamine specific groups (–RC=N–), usually formed by condensation of ammonia and reactive carbonyl compounds. Over the past few decades, Schiff bases and their complexes have been the focus of increasing attention due to their pharmacological activities, which include anti-inflammatory, anti-microbial, anti-viral and anti-tumor effects^8,20–22^. Previous studies have confirmed significant anti-tumor effects of Valdien, which belongs to the family of Schiff bases, against colon cancer and human non-Hodgkin’s lymphoma. However, Valdien has poor water solubility, limiting its clinical application, and its effects against breast cancer as well as the underlying mechanisms have not been elucidated. Therefore, we synthesized Schiff base ligands from o-vanillin derivatives, identified one with high water solubility (VALD-3), and investigated its anti-tumor effects and potential mechanisms of action in vivo and in vitro.

To measure the cytotoxic effects of VALD-3 against MCF-7 and MDA-MB-231 cells, we employed the MTT assay. The results showed that VALD-3 exerted significant time- and dose-dependent anti-proliferative effects against MCF-7 and MDA-MB-231 cells, indicating that VALD-3 had the ability to inhibit the growth of breast cancer cells in vitro. At the concentration 40 mg/l, VALD-3 reduced the viability of MCF-7 and MDA-MB-231 cells to only 24.01 ± 5.87% and 9.69 ± 1.31% of the control values, respectively. These results indicate that VALD-3 inhibits cell proliferation and induces cytotoxicity.

Apoptosis is a form of induced cell death triggered by multiple signaling pathways. It involves regulation at the gene level and results in the orderly and efficient removal of damaged cells^23,24^. Studies have demonstrated that apoptosis can be initiated by engagement of a death receptor (the so-called extrinsic pathway), or by the mitochondrial (or intrinsic) pathway^25^. The activation of caspases ultimately results in morphological and biochemical changes common to both the death receptor and mitochondrial pathways^26,27^. The best characterized death receptors of the extrinsic pathway are Fas and TNFR1, in which caspase 8, caspase 3 and other downstream caspases are activated, triggering the apoptotic cascade^28^. An intracellular protein–protein interaction domain, called the death domain (DD), structurally defines the death receptors and is closely related to the induction of apoptosis signals^29^. In the mitochondrial-mediated pathway, different stress conditions cause cytochrome *c*, an apoptosis-inducing factor, to be released from mitochondria into the cytosol. Cytochrome *c* can then form the apoptosome by binding to cytosolic Apaf-1 (apoptosis protease activating factor-1), activating caspase 9 and downstream effectors (caspase-8, caspase-3 and PARP), and ultimately triggering auto-activation and apoptosis^30,31^. In the current study, Hoechst 33258 staining showed that VALD-3 induced apoptotic body formation in MCF-7 and MDA-MB-231 cells. In addition, we quantified early and late apoptosis by flow cytometry to determine the effects of VALD-3 on breast cancer cells. Consistent with the Hoechst 33258 staining results, we found that VALD-3 induced apoptosis of breast cancer cells. VALD-3 seems to have a stronger apoptosis-inducing effect against triple-negative breast cancer MDA-MB-231 cells than against MCF-7 cells. Western blotting results showed that treatment of breast cancer cells with VALD-3 resulted in cleavage/activation of cytochrome *c*, caspase-8, caspase-3 and PARP.

Bcl-2 family proteins are pivotal regulators of apoptosis, and their anti-apoptotic and pro-apoptotic members play vital roles in the mitochondrial pathway^32^. Whereas anti-apoptotic proteins (Bcl-2, Bcl-xL) can block apoptosis by inhibiting their pro-apoptotic counterparts through protein–protein interactions, pro-apoptotic proteins (Bax, Bad) facilitate this process by promoting the mitochondrial release of cytochrome-*c*, ultimately resulting in the cleavage of critical cellular proteins^30,33–35^. Our results showed that VALD-3 upregulated the levels of pro-apoptotic Bax and Bad proteins while simultaneously downregulating the levels of anti-apoptotic Bcl-2, Bcl-xl proteins. On the other hand, X-linked inhibitor of apoptosis protein (XIAP) and survivin are two factors showing the strongest apoptosis-inhibitory effects of the known inhibitor of apoptosis proteins (IAPs). IAPs are endogenous proteins that can strongly inhibit apoptosis triggered by various factors. Studies have shown that XIAP is a crucial therapeutic target in cancer^36,37^. We found that the expression of Survivin and XIAP protein was markedly reduced in VALD-3-treated MCF-7 and MDA-MB-231 cells. These results indicate that VALD-3 induces apoptosis of MCF-7 and MDA-MB-231 cells partly through the mitochondrial apoptotic pathway.

It is widely accepted that cell cycle arrest can induce apoptosis. Many anticancer agents alter the regulation of the cell-cycle machinery, leading to cell cycle arrest at any phase and inhibition of tumor cell growth^38,39^. Cell cycle regulation is mediated by a combination of cyclins, CDKs and CDK inhibitors (CDKI). Cyclin D specifically regulates the G1/S phase, and Cyclin D overexpression accelerates progression from G0/G1 to S. Cyclin B and CDK1, two specific regulators of the G2/M phase, interact with each other and form the maturation promoting factor (MPF)^40–42^. The S phase arrest induced by VALD-3 in MCF-7 and MDA-MB-231 cells may be linked to downregulation of Cyclin D. On the other hand, based on flow cytometric analysis, the G2/M arrest of MCF-7 cells may be related to Cyclin B and CDK1 downregulation. However, it is surprising that although VALD-3 downregulated cyclin B1 and CDK1 in MDA-MB-231 cells, the induction of G2/M phase arrest was not observed. Collectively, these results indicate that inhibition of cell cycle progression may underlie the anti-cancer effects of VALD-3 against breast cancer cells.

The Wnt pathway is one of the most important pathways regulating the development of breast cancer^43^, andβ-catenin is a key regulator of the Wnt pathway. This pathway is not active in normal mature cells. When the Wnt pathway is activated, β-catenin is dephosphorylated and enters the nucleus to bind to the transcription factor TCF/LEF, thereby turning on the transcription of downstream c-myc and other target genes^44^. For this reason, we explored the effects of VALD-3 on the Wnt/β-catenin pathway in breast cancer cells by Western blotting. We found evidence that β-catenin was significantly downregulated, and transcription factor TCF/LEF and downstream proteins were also reduced, indicating inactivation of the Wnt/β-catenin signaling pathway.

However, anti-tumor effects of VALD-3 in vitro does not necessarily mean that it will also have anti-tumor effects in vivo. Therefore, we established an MCF-7 tumor xenograft model to evaluate the anticancer effects of VALD-3 in vivo. We found that VALD-3 at 20 mg/kg inhibited tumor growth with a potency similar to cisplatin. We also evaluated the expression of β-catenin and its downstream proteins, XIAP and Survivin, in tumor tissues. The results showed that VALD-3 lowered the expression of XIAP, Survivin and the Wnt/β-catenin signaling pathway, in agreement with the in vitro results. In addition, the Tunel assay showed important differences between the control and experimental groups. VALD-3 (20 mg/kg/d) obviously induced apoptosis of breast cancer cells. The survival analysis showed that VALD-3 prolonged the survival of tumor-bearing mice when compared with the control group. Moreover, H&E staining showed that VALD-3 markedly suppressed tumor growth with no clear signs of damage to the major organs. Therefore, VALD-3 showed marked tumor inhibitory effects and low toxicity in vivo. These results indicate that it may be a good anticancer drug candidate.

In conclusion, this study provides evidence that VALD-3 inhibits the proliferation of breast cancer cells in vitro and in vivo. Our study demonstrated that VALD-3 reduced cell viability and colony formation, and induced S phase arrest and cellular apoptosis. In addition, modulation of the Wnt/β-catenin signaling pathway was involved in the VALD-3-induced apoptosis of MCF-7 and MDA-MB-231 cells. Collectively, these results demonstrate the anti-tumor effects of VALD-3 and its probable mechanisms of action. Therefore, VALD-3 may be an alternative strategy for the treatment of breast cancer.

## Supplementary Information

Supplementary figures.
