# Supplementary material for: VALD-3, a Schiff base ligand synthesized from o-vanillin derivatives, induces cell cycle arrest and apoptosis in breast cancer cells by inhibiting the Wnt/β-catenin pathway
Source: Sci Rep. 2021 Jul 22;11:14985. doi: 10.1038/s41598-021-94388-x (PMC8298535; doi:10.1038/s41598-021-94388-x)
Supplement: Supplementary file 1 — Supplementary figures. [file 41598_2021_94388_MOESM1_ESM.docx]

Title: VALD-3, a Schiff base ligand synthesized from o-vanillin derivatives, induces cell cycle arrest and apoptosis in breast cancer cells by inhibiting the Wnt/β-catenin pathway

Authors’ names: Hongling Li^1^, Chunyan Dang^1^, Xiaohui Tai, Li Xue, Yuna Meng, Shuping Ma, Jing Zhang

Authors’ affiliations: Division of Oncology, Gansu Provincial Hospital, Lanzhou 730000, Gansu, PR China

XIAP
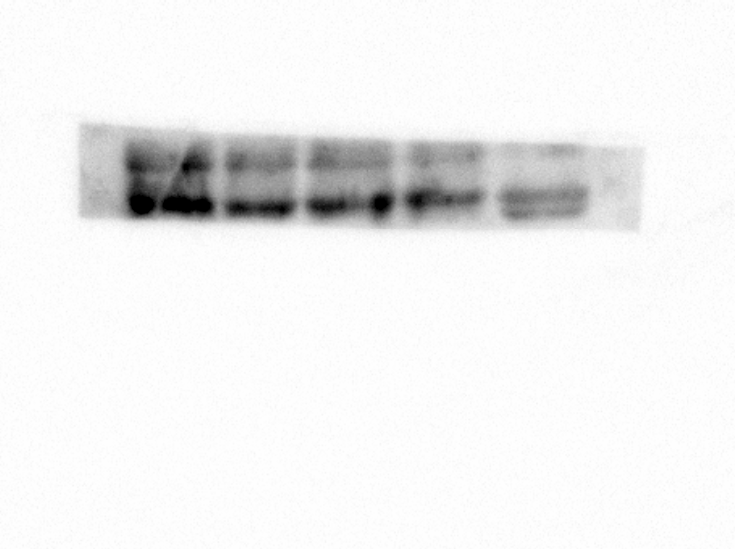

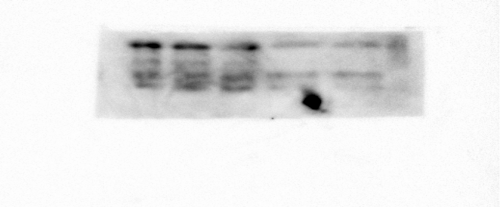


Survivin
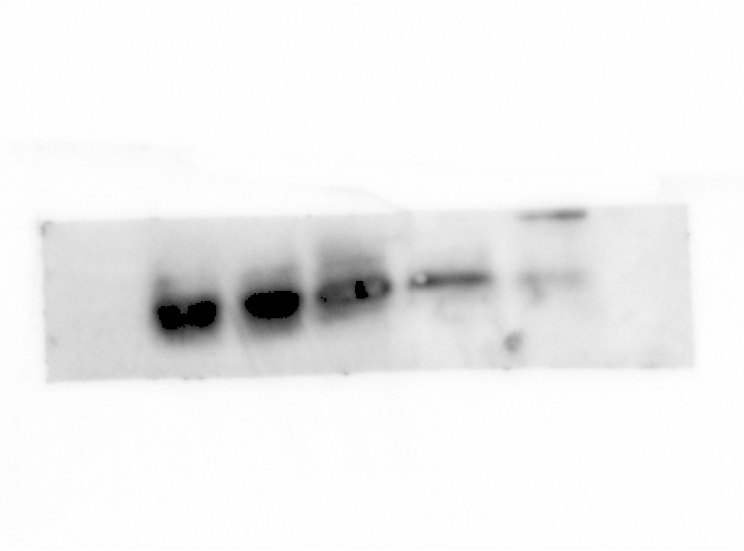

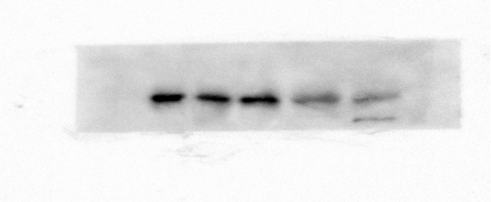


Bcl-xl
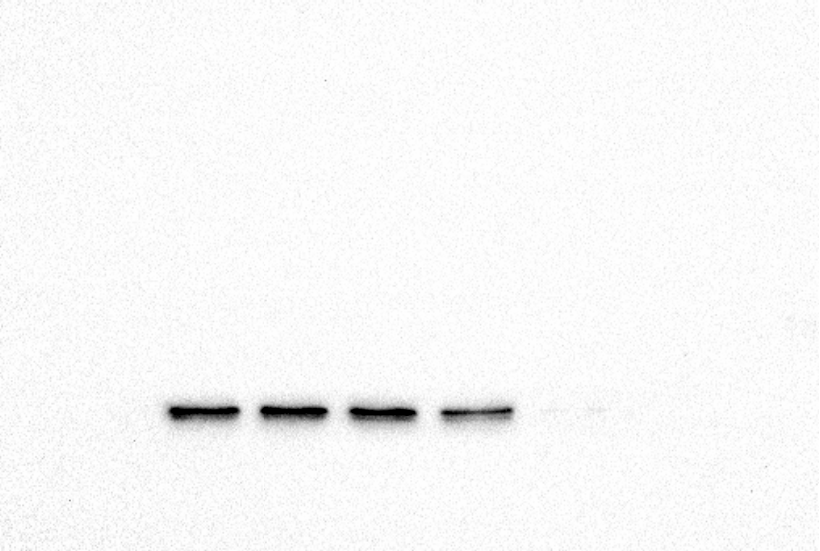

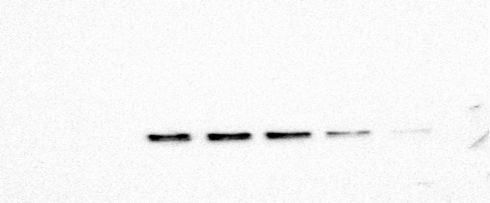


Bcl-2
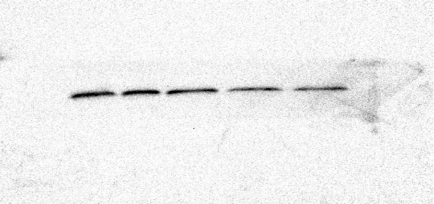

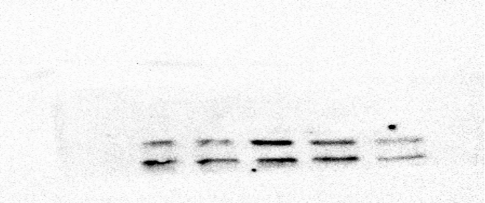


Bax
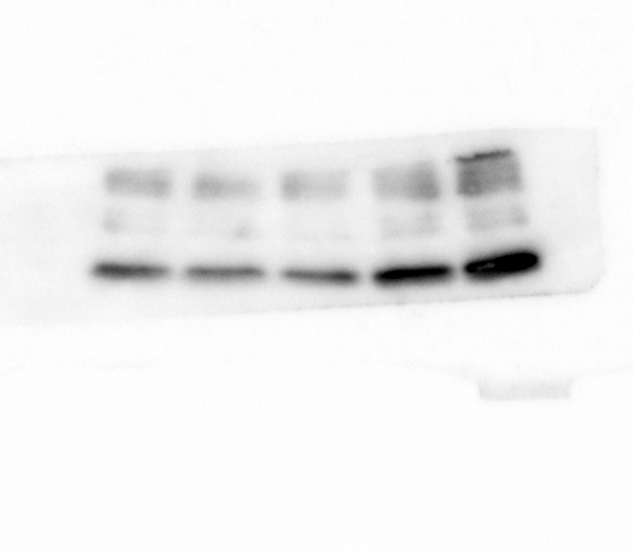

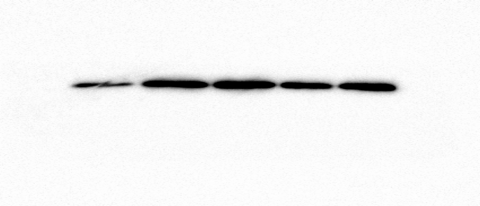


Bad
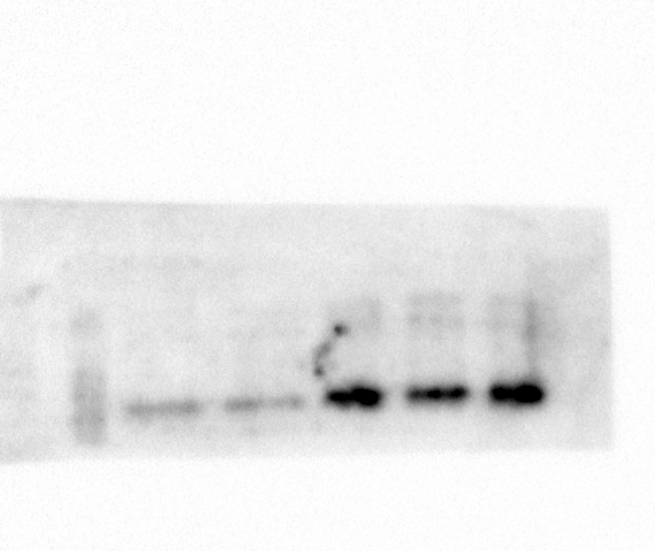

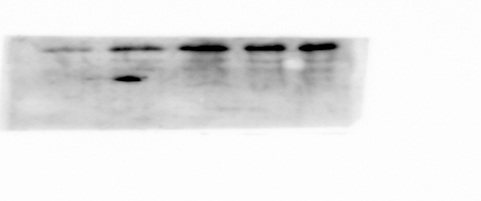


Β-actin
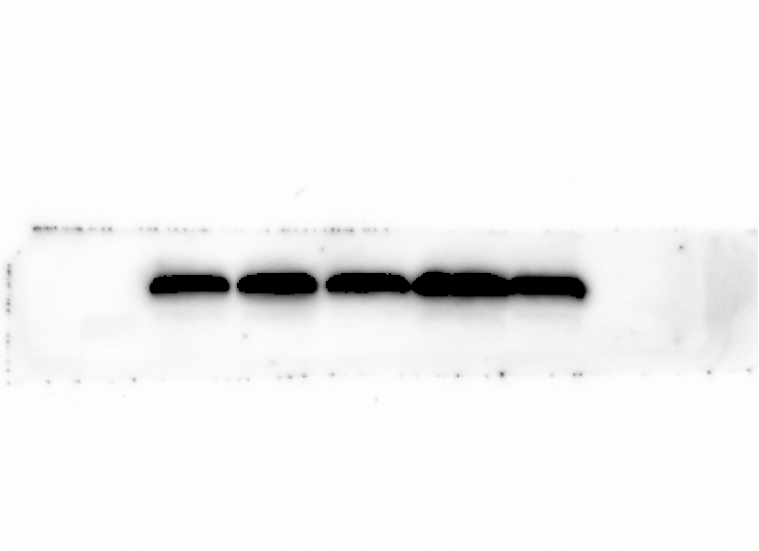

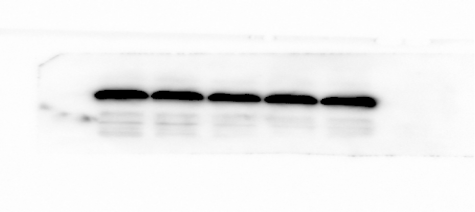


MCF-7 cells MDA-MB-231

PARP
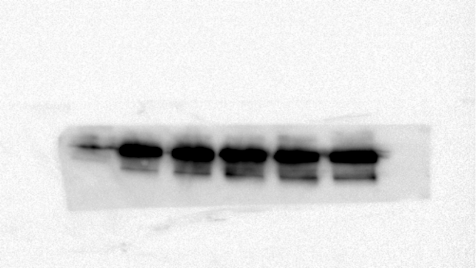

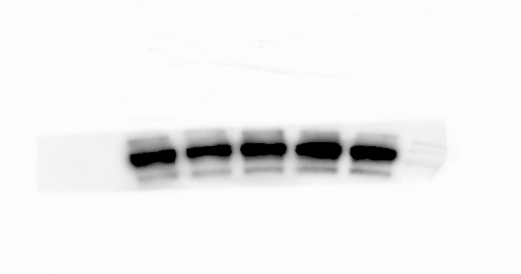


Caspase-8
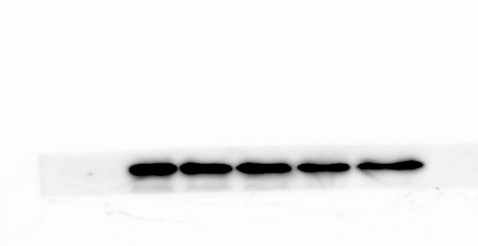

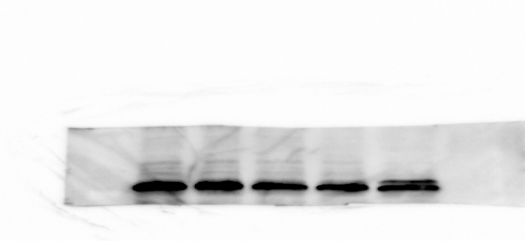


Caspase-3
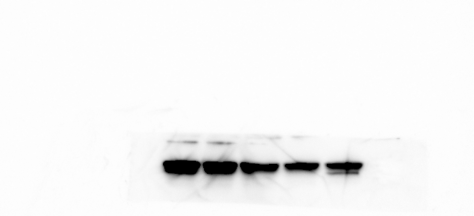

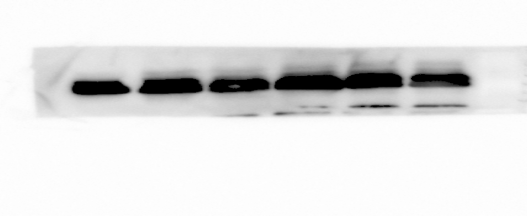


Cyto-c
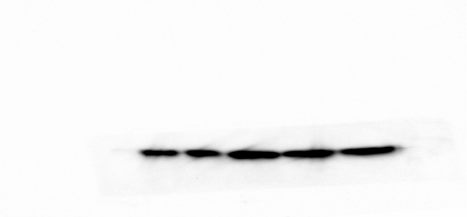

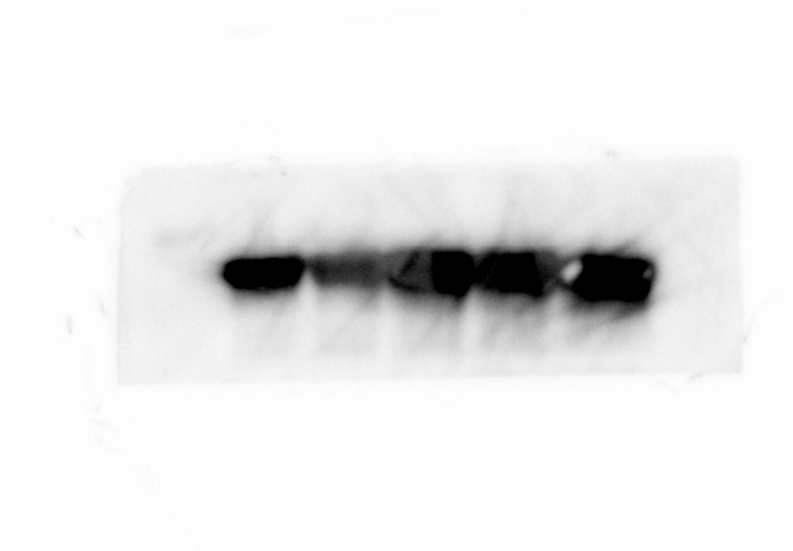


Β-actin
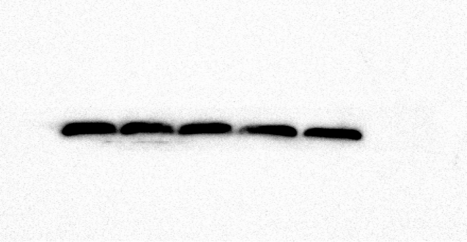

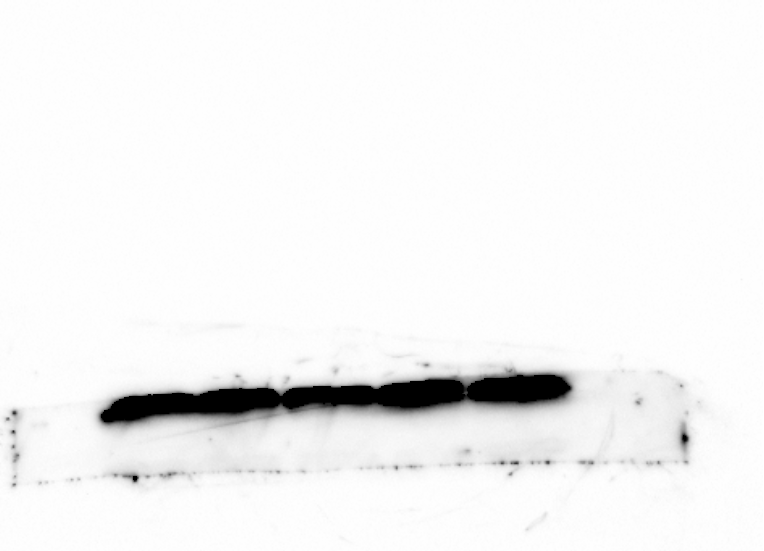


MCF-7 cells MDA-MB-231

CD44
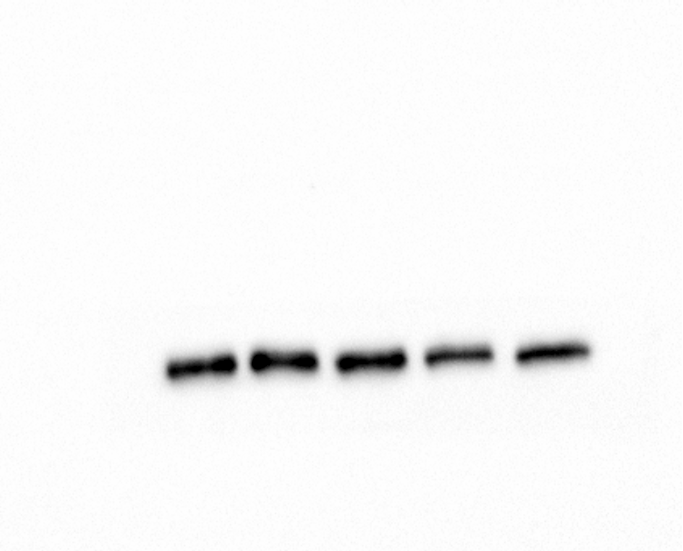

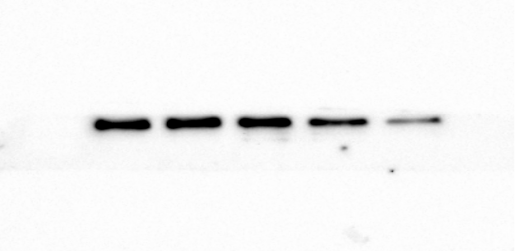


C-met
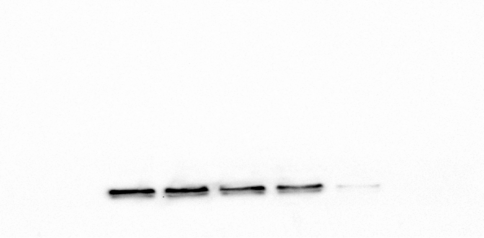

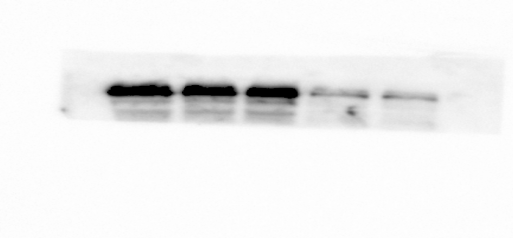


LEF-1
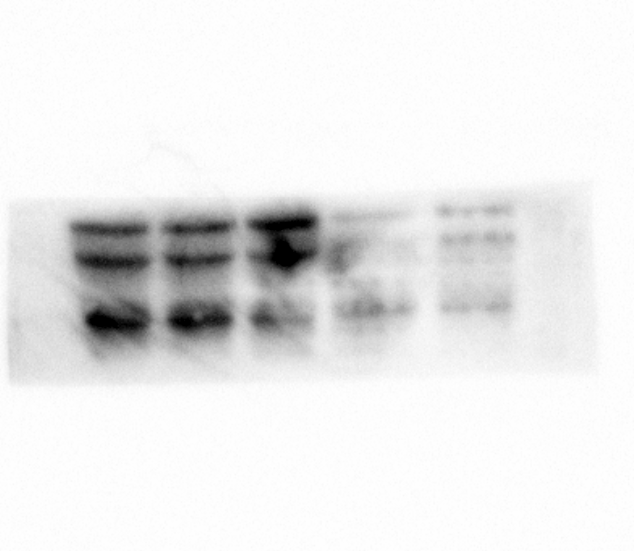

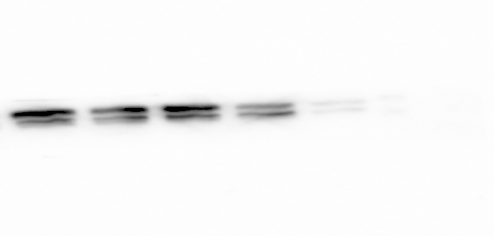


C-myc
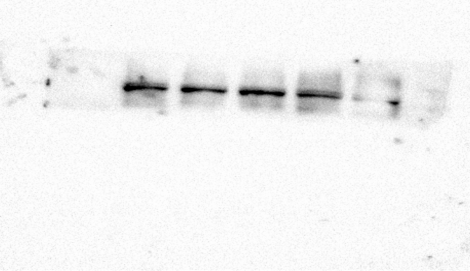

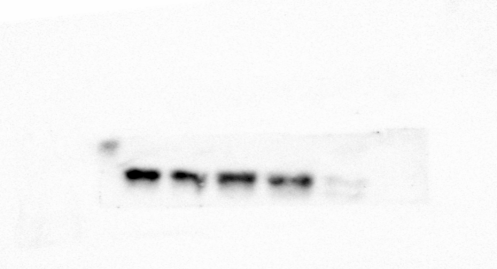


Β-catenin
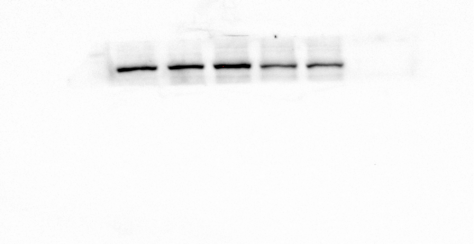

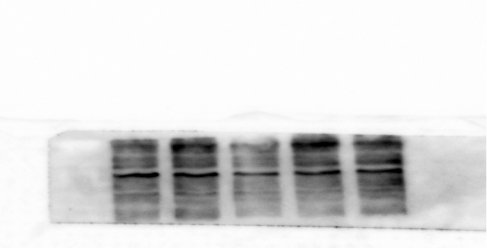


Β-actin
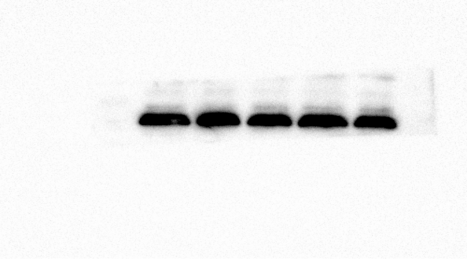

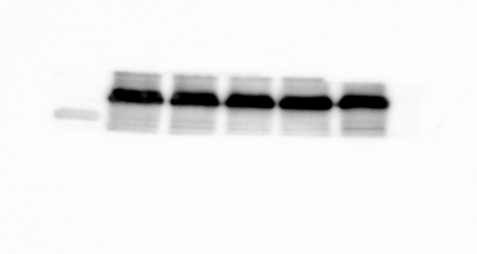


MCF-7 cells MDA-MB-231

CyclinB1
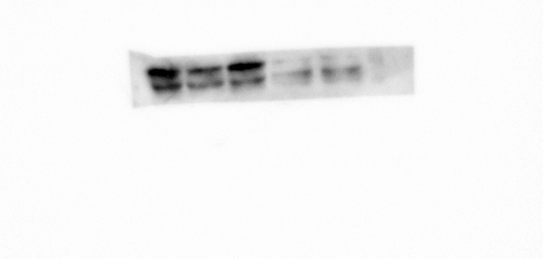

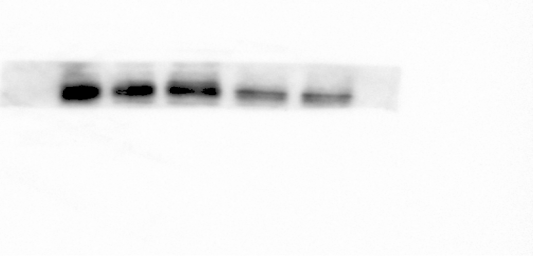


CyclinD1
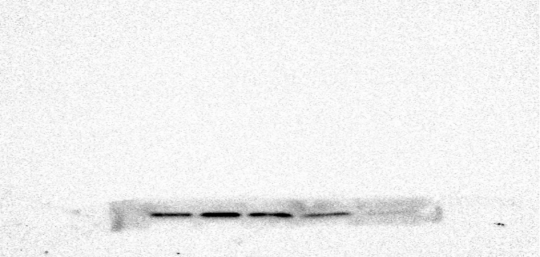

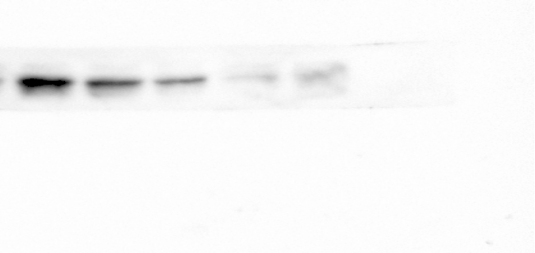


CDK1
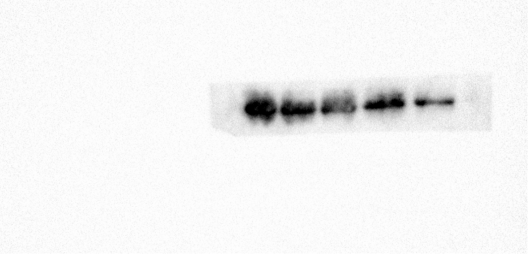

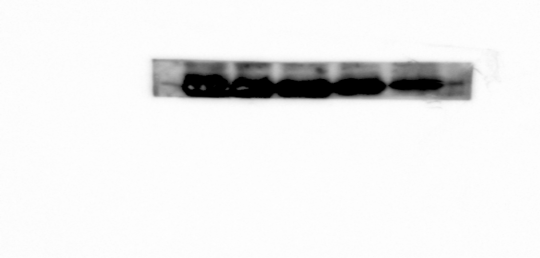


Β-actin
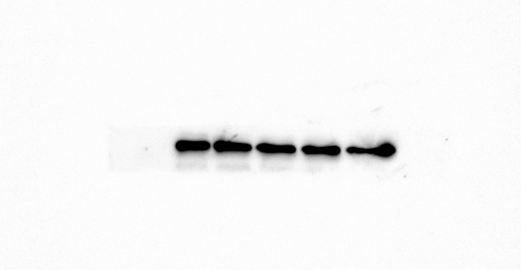

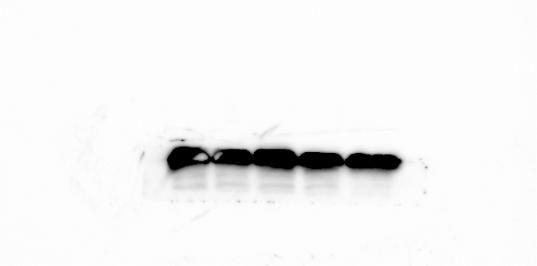


MCF-7 cells MDA-MB-231
